# Sensitivity Analysis of Open-Top Cartons in Terms of Compressive Strength Capacity

**DOI:** 10.3390/ma16010412

**Published:** 2023-01-01

**Authors:** Damian Mrówczyński, Tomasz Gajewski, Tomasz Garbowski

**Affiliations:** 1 Doctoral School, Department of Biosystems Engineering, Poznan University of Life Sciences, Wojska Polskiego 28, 60-637 Poznań, Poland; 2 Institute of Structural Analysis, Poznan University of Technology, Piotrowo 5, 60-965 Poznań, Poland; 3 Department of Biosystems Engineering, Poznan University of Life Sciences, Wojska Polskiego 50, 60-627 Poznań, Poland

**Keywords:** sensitivity analysis, corrugated board, trays, open-top packaging

## Abstract

Trays in which fruit and vegetables are transported over vast distances are not only stored in extreme climatic conditions but are also subjected to long-term loads. Therefore, it is very important to design them correctly and select the optimal raw material for their production. Geometric parameters that define the shape of the packaging may also be optimized in the design process. In this work, in order to select the most important parameters that affect the load capacity of a tray, sensitivity analysis was used. A sensitivity analysis is often the first step in the process of building artificial-intelligence-based surrogates. In the present work, using the example of a specific tray’s geometry, the selection of starting parameters was carried out in the first step, based on the Latin hypercube sampling method. In the next step, local sensitivity analyses were performed at twenty selected starting points of the seventeen-dimensional space of the selected parameters. Based on the obtained results, it was possible to select the parameters that have a significant impact on the load capacity of the tray in the box compression test and whose influence is negligible or insignificant.

## 1. Introduction

Open-top, corrugated board cartons have become one of the most popular ways to transport fruits and vegetables. Long-distance transport applies to raw food products such as tomatoes, peppers, bananas, apples, lemons, and many more. The natural material of paper favors the long-distance transport of such products, especially when moisture and temperature changes occur frequently during transport. Corrugated cardboard packaging is also perceived by consumers as ecologically friendly, and scientific studies have confirmed that customers are willing to pay more for environmentally sustainable packaging [1,2,3]. For instance, for decades bananas, have been effectively transported in cartoon boxes. In 2020, 21.5 million tons of bananas were exported, from which about 16.5 million tons were exported from Africa. The European Union and the United States are the biggest importers, accounting for 26% and 21% of the share in global imports in 2020 [4]. The ventilation conditions and the maturation of bananas during transport are crucial throughout the production process.

**Figure 1:**
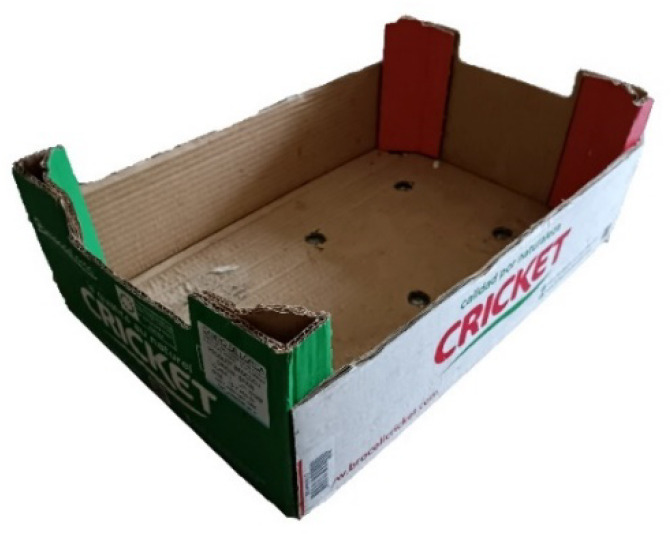
Selected example of an open-top box for the transport of fruits or vegetables.

Open-top, corrugated board cartons prevail over other types of packaging if the particular ventilation conditions are ensured. The ventilation holes allow air to circulate inside the packaging, which also enables water to evaporate from the inside of the container, which, in turn, protects the raw food products from undesirable moisture. Superior air flow in the packaging facilitates the disposal of ethylene, which causes the fruit to ripen as an aging-stimulating hormone. Therefore, on the one hand, the better the ventilation, the better the fruit’s condition, while on the other hand, the more ventilation holes, the lower the packaging strength (i.e., less material to bear the loads) [5,6,7]. Both objectives are contradictory, although the appropriate location of the ventilation holes can significantly reduce the loss of the load-bearing capacity of the packaging and, at the same time, maximize air flow. In Figure 1, an example of an open-top carton for the transport of fruits/vegetables is presented. Optimizing the use of material to maximize the efficiency of the packaging (regardless of the measure, e.g., compressive strength, resistance to random vibrations, ventilation of products, etc.) is, and will always be, a significant problem in the field of packaging design [8].

Open-top, corrugated board cartons constitute a very specific form of packaging with a complex structure, which is significantly different from typical flap packaging such as F0201 according to FEFCO codes [9]. Due to the complexity of this type of packaging, the use of known analytical formulas to determine its compression load capacity is inadequate [10,11,12,13,14,15,16]. The popular formulas are adequate for simple flap boxes. Many geometrical parameters for determining the shape of open-top packaging for fruits/vegetables make it difficult, and even impossible, to derive the analytical formula for computing the compressive strength of this type of packaging. Therefore, to avoid relying on the experience of the designer, numerical tools for the estimation of strength should be adopted. However, building a model for complex packaging is not a trivial task; it requires experience in numerical modelling. In this regard, the utilization of artificial neural networks (ANNs) preceded by a numerical study could be the solution, for instance, as performed in [17,18]. Numerical methods and ANNs are used effectively in similar problems in the food/packaging industry [19,20,21,22] and others [23,24]. An ANN model must be versatile enough to cover a moderately wide range of possible designs. ANNs can be efficient, even for a large number of parameters; however, many parameters require numerous pre-processing computations to prepare input data for the ANN’s training, testing, and validation [25]. The increase in the computational cost with the increasing number of parameters is non-linear.

A good practice in building an artificial neural network is to analyze the sensitivity of potential input parameters to the network so that the output argument of the network depends meaningfully on the input arguments. It is recommended that the ANN inputs (i.e., all the model parameters) that have proven to exert a very low level of influence on the ANN’s output (i.e., BCT value) are removed from the model to limit the pre-processing cost. A reliable sensitivity analysis for open-top corrugated board cartons for fruits and vegetables is not available in the scientific literature; therefore, addressing this gap is the main aim of the paper. There are papers that analyze the role of horticultural cartons’ vent hole design on cooling efficiency and compression strength [26,27,28]; however, this constitutes research into a different type of cartoon.

In this paper, we will indicate the sensitivity of an open-top cartoon in respect to its main geometrical parameters. We will point out which of the parameters may be omitted while building the ANN network to estimate the compression strength of the open-top box in order to limit the computational cost of ANN input data. Resultantly, the parameters that seem crucial for the corrugated board packaging industry do not have a significant impact on the compression strength of the packaging.

## 2. Materials and Methods

### 2.1. Geometric Parametrization of Open-Top Boxes

**Figure 2:**
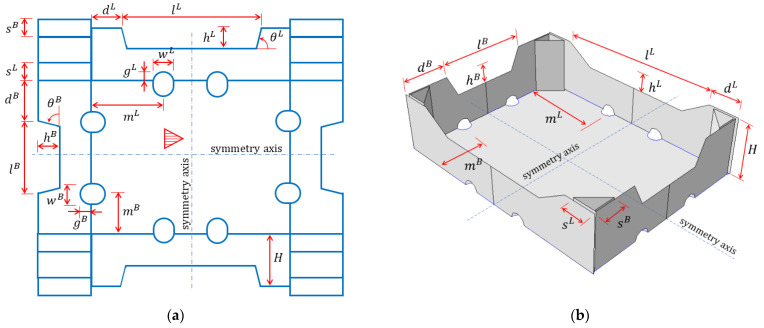
Parametric model used in the study: (**a**) 2D grid and (**b**) 3D scheme (for clarity, $w^{L}$, $g^{L}$, $w^{B}$, $g^{B}$, $\theta^{L},$ and $\theta^{B}$ parameters are not shown here).

There are many types of trays for fruit and vegetables in the corrugated carboard packaging offered by its respective industry, but there are some constructions that have repeatable folding and similar dimensions. Manufacturers often have their own packaging designs selected on the basis of their own experience. As part of this work, a type of tray that is relatively popular in the market was selected. The geometry of such packaging is shown in Figure 2 as 2D drawing and in 3D view. In this type of packaging, the sidewalls fold vertically, and the elements of the shorter walls form rectangular triangles reinforcing the corners. One of the sides of the triangular corners is glued to the longer sidewalls, which ensures the rigidity of the corners. Moreover, on the bottom edges of the tray, there are circular or oval ventilation holes, usually two for each edge. In addition, there are trapezoidal folds at the tops of the sidewalls (one for each wall). These folds are bent down and glued to strengthen the sidewalls (not shown in the drawings).

Based on the selected packaging design, over a dozen geometric parameters were defined, which are labelled in Figure 2. The selection criteria were as follows: (i) the suspected significance of the impact on the box compression test (BCT) value and (ii) its importance in terms of wicking or other functions of packaging. These selected design parameters are as follows:

- $d^{L}$, half of the horizontal length of the non-folded part of the longer sidewalls;
- $d^{B}$, half of the horizontal length of the non-folded part of the shorter sidewalls;
- H, the height of the stiffening triangles and the box;
- $l^{L}$, the width of the trapezoidal folds on the longer sidewalls;
- $h^{L}$, the height of the trapezoidal folds on the longer sidewalls;
- $l^{B}$, the width of the trapezoidal folds on the shorter sidewalls;
- $h^{B}$, the height of the trapezoidal folds on the shorter sidewalls;
- $s^{L}$, the length of the sides of the stiffening triangles on the longer sidewalls;
- $s^{B}$, the length of the sides of the stiffening triangles on the shorter sidewalls;
- $w^{L}$, the width of the edge holes on the longer sidewalls;
- $g^{L}$, the height of the edge holes on the longer sidewalls;
- $m^{L}$, the dist. of the edge holes on the longer walls from the shorter walls to its axis;
- $w^{B}$, the width of the edge holes on the shorter sidewalls;
- $g^{B}$, the height of the edge holes on the shorter sidewalls;
- $m^{B}$, the dist. of the edge holes on the shorter walls from the longer walls to its axis;
- $\theta^{L}$, inclination of the arms of the trapezoidal folds on the longer sidewalls;
- $\theta^{B}$, inclination of the arms of the trapezoidal folds on the shorter sidewalls.

Note that the $d^{L}$ and $d^{B}$ modifications change the in-plane dimensions of the box model (because, in this case, $l^{L}$ and $l^{B}$ are constant), while the $l^{L}$ and $l^{B}$ modifications do not change these dimensions (due to simultaneous changes of $d^{L}$ and $d^{B}$).

**Figure 3:**
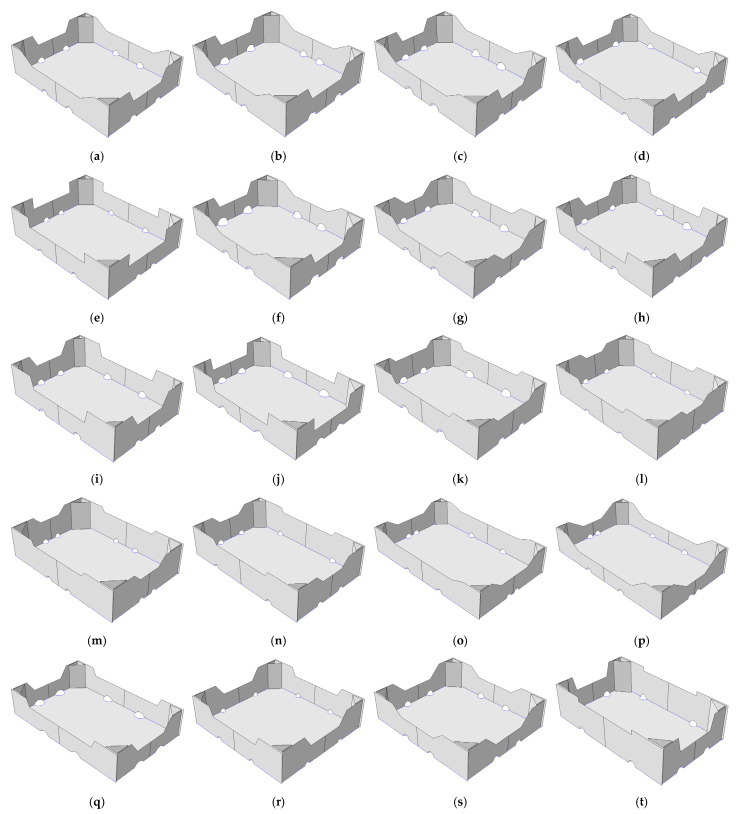
Cases of selected open-top boxes for the storage of fruits. (**a**–**t**) selected twenty carton geometries.

After determining the box’s design parameters, listed above, a numerical algorithm was created using MATLAB software in order to automatically generate the FE model in Abaqus FEA, which is capable of simulating a box compression test. After creating the algorithm, the twenty sets of design parameters were determined according to a Latin Hypercube Sampling (LHS) strategy. One of the examples of LHS algorithm was published by Jin et al. [29]. LHS was used to explore the space of seventeen design parameters most effectively in practically applicable ranges. The use of such a strategy was dictated by the need to build a dataset concerning the sensitivity of the system in a global sense. Exploration of the parameter space in selected 20 locations allows one to understand the relationship between the sensitivity of a given parameter and its initial value. The final sets of design parameters are presented by numbers in Table 1 and graphically by box designs in Figure 3. It is worth mentioning that the presented approach allows for a very even sampling of the space of all seventeen parameters of the model, whose sensitivity to minor perturbations in these parameters at different points in the space may be different. Therefore, this approach allows for the acquirement of averaged responses in the form of sensitivities to all model parameters in the full range of parameters’ space.

### 2.2. Finite Element Model of Open-Top Boxes

**Figure 4:**
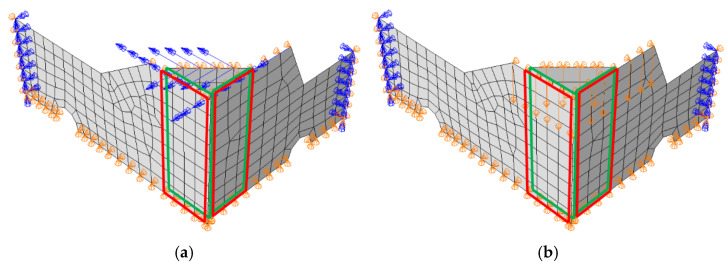
Finite element model of open-top boxes for transport of fruits with boundary conditions and mesh (the red and green rectangles indicate where the vertical faces are in contact): (**a**) buckling step; (**b**) compression step.

Numerical models were created in commercial software FE (Abaqus Unified FEA software [30]) to simulate box compression test (BCT) of open-top boxes. In order to reduce the number of finite elements and shorten computational time, only 1/4 of the box was modeled. The bottom of the packaging was omitted because the bottom does not contribute to the compression-related load-bearing capacity. To ensure robust behavior of the model, the above-mentioned simplifications have been replaced with appropriate boundary conditions (see Figure 4). Symmetrical boundary conditions on the two sidewalls were applied and the vertical displacements of the top edges of the box panels were blocked. Out-of-plane displacements of the bottom and top edges of the panels were also blocked. The analysis consists of two computational steps. In the first step, a buckling analysis was performed in order to compute the first mode, which was applied as imperfection to the model, and in the second nominal step, the packaging was loaded by applying vertical displacement on the top edges. In Figure 4, the boundary conditions for both steps are shown. The figure also presents the corner panels (green color) that are glued to the sidewall fragments (red color) in order to assemble the packaging. In numerical model, this connection of panels has been mapped by special numerical techniques, so-called ‘tie connection’, which ensures the integrity (continuity of displacements) of the structure between two parts considered.

The analysis was performed for three different corrugated boards that were modeled as linear elastic orthotropic material with Hill plasticity [31]. In Table 2, the parameters of three materials used in the model are given. The material data were determined by the BSE System via FEMAT [32] from mechanical tests of corrugated board samples. Samples were prepared in laboratory and conditioned in a climate chamber. For each test, 10 samples were used to acquire statistically representative material data. In the first column of Table 2, the grade symbol represents the type of the wave and the grammage of the cardboard in $g/m^{2}$. Columns 2–7 contain elastic material parameters: $E_{1}$ and $E_{2}$ are the moduli of elasticity, $\nu_{12}$ is the Poisson’s ratio, $G_{12}$ is the in-plane shear stiffness, $\mathrm{and}\;G_{13}$ and $G_{23}$ are the transverse shear stiffnesses. The last two columns contain plastic parameters of the material; $\sigma_{0}$ is the initial yield stress and $R_{11}$ is the yield stress ratio in the machine direction of the corrugated cardboard.

For each material, 20 boxes with dimensions shown in Table 1 were analyzed. Each of the parameters from Table 1 for each grade and box design was subjected to a 1% perturbation. This means that 18 analyses were performed for each of the 20 geometries (one reference case and 17 analyses with one parameter changed). In total, this yielded 1080 numerical models (3 materials × 20 packaging designs × 18 analyses). In each model, 4-node quadrilateral shell elements with full integration, named S4 according to [30], were used, and they were completed with 3-node triangular shell elements with full integration, named S3 according to Abaqus FEA. A global mesh size equal to 10 mm was assumed, which resulted in a different number of nodes and elements for each geometry case. For example, for the first case, 373 elements (368 quadrilateral elements and 5 triangular elements) and 437 nodes were obtained, as shown in Figure 4. The choice of such a finite element dimension was based on the observations made in our previous studies [9,33,34], as well as the validation procedure presented in the next section, which was carried out to verify the computational models and commercial tools used.

### 2.3. Model Validation

**Figure 5:**
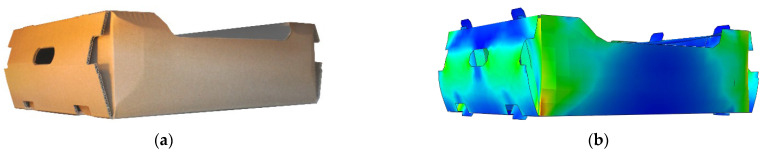
Model validation: (**a**) the box sample during the test and (**b**) its numerical counterpart at maximal strength (maximum reaction force value).

The finite element model used in this research (see Section 2.2) was validated through experimental research. Namely, ten samples of open-top cartons for the storage of vegetable or fruits were manufactured and tested in a mechanical press in order to compare the experimental results of BCT with the numerical prediction according to the computational approach used in the study. In the computational model for validation, the boundary conditions, mesh (element size, element type, etc.), constitutive law, and two-step strength analysis (buckling followed by static analysis) were the same as those described in Section 2.2. The sample of open-top carton for validation is shown in Figure 5a and its numerical model geometry is depicted in Figure 5b. In Figure 5b, the deformed box obtained at maximal compression force was confronted with its numerical counterpart (see Figure 5b). It is visible that the deformation modes are in good agreement. Moreover, if the compression strengths obtained are compared, the numerical prediction was burdened with 6.4% error compared to the average strength obtained from tests.

Notably, in this study, the sensitivity of the model to the size of the finite element mesh was also ascertained. It was concluded from the analyses that the models in which elements with dimensions of about 15 mm were used were characterized by slightly increased stiffness in the elastic buckling phase; however, in the non-linear phase, the use of smaller elements (e.g., 5 mm) did not increase the precision of the calculations. Therefore, in the model used for the final verification, a grid with elements of about 10 mm was used.

### 2.4. Sensitivity Analysis

In this paper, the sensitivity analysis of BCT was performed for 20 open-top carton geometries. In each case, the parameters of the model were the dimensions of the packaging (see Figure 2), which were collected in vector x. The BCT value for the selected set of parameters can be denoted as h(x). Then, by small perturbations of the i-th parameter $\Delta x_{i}$, it is possible to calculate the change in the investigated quantity $h\left(x\pm e_{i}\Delta x_{i}\right)$, where $e_{i}$ is the unit vector of the i-th parameter in the parameter space. Determination of the numerical gradient using, e.g., the central difference, allows one to obtain the sensitivity of the compressive strength to the change in the considered parameter, according to the following formula:

(1) $$s=\frac{h\left(x+e_{i}{\Delta x}_{i}\right)-h\left(x-e_{i}\Delta x_{i}\right)}{2\Delta x_{i}}\frac{x_{i}}{h\left(x\right)}\;.$$

The performed analysis is a non-local sensitivity analysis. This means that the computations were carried out for many points in the parameter space (20 packaging designs) to build a dataset concerning the entire range of space, and not just locally at one specific point. In this paper, the approach to compute the sensitivity by Equation (1) is similar to the one used in [33,34].

## 3. Results

**Figure 6:**
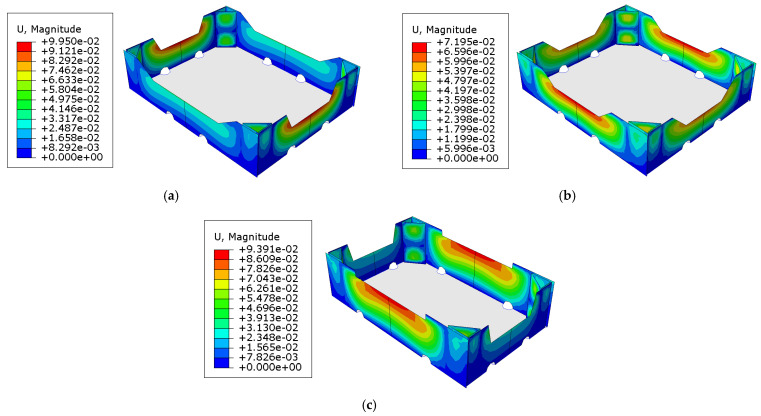
Selected buckling modes used for nominal strength analysis using the finite element method: (**a**) case 1, (**b**) case 19, and (**c**) case 20.

First, the material data used to model the corrugated cardboard were acquired. Then, the geometry of the packaging together with the material data were used to build the models in the FE software. Next, for the created models, buckling analyses were performed, from which maps of displacement of individual panels were obtained in order to determine the initial imperfections of the model to be used in further computations. In order to calculate the buckling modes, the vertical displacement on the top edges of the box was used instead of the loads. In Figure 6, the displacements calculated for examples of cases 1, 19, and 20 are presented.

**Figure 7:**
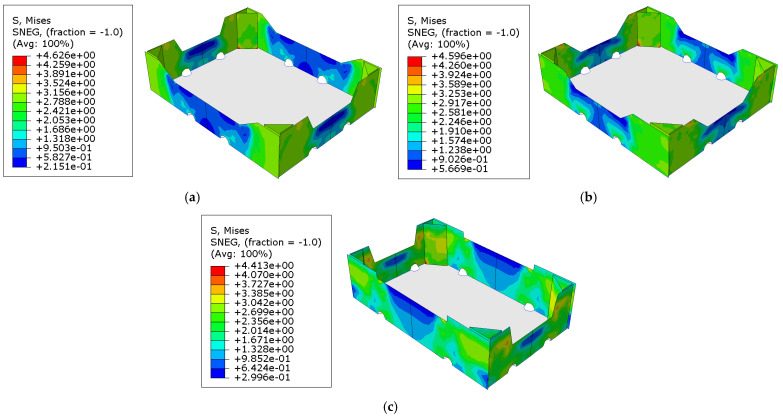
Stress distributions at the moment of reaching the compression load capacity of open-top boxes for storage of fruits: (**a**) case 1, (**b**) case 19, and (**c**) case 20.

The second step of the numerical analysis was the compression of the packaging. This made it possible to obtain the force–displacement relationship and identify the compression load capacity of the box. In Figure 7, the effective stresses of the Huber–Mises-Hencky distributions for selected cases of open-top boxes for the storage of fruits are shown. The bottom of the packaging was not modeled but was included in the computations using appropriate boundary conditions. In Figure 6 and Figure 7, the bottom of the box is added for visualization purposes.

As described at the end of Section 2.2, 20 cases of packaging geometry were analyzed (see Table 1). Considering a specific type of geometry, first, the reference value of the compression strength capacity was calculated. Then, each parameter was perturbed individually by 1% and the load capacity of the box was computed. All these computations made it possible to obtain 360 results for each material and a total of 1080 values of the load capacity. Based on the results obtained, the sensitivities of each of the 17 parameters were calculated for 20 packaging geometries and 3 types of corrugated board. All sensitivities were calculated from Equation (1), where h(x) is the load-bearing capacity with respect to top-to-bottom compression. In Table 3, examples of the sensitivities computed for B-840 corrugated cardboard are presented.

**Figure 8:**
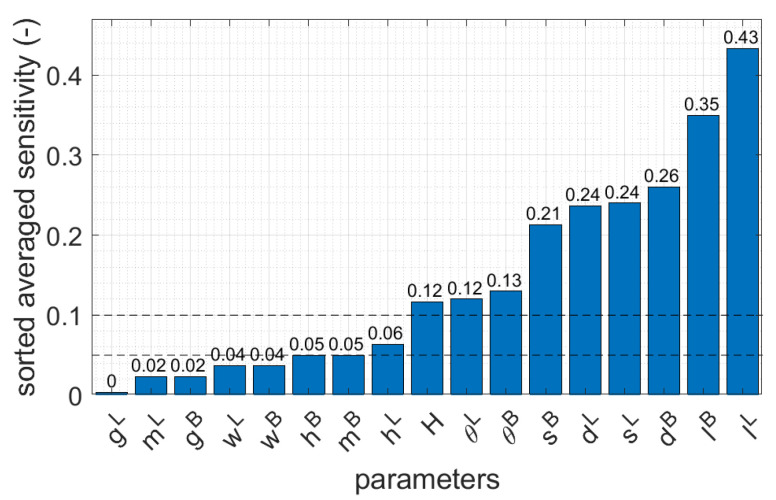
The averaged values of parameter sensitivities for three corrugated cardboards considered (sorted in ascending order).

**Figure 9:**
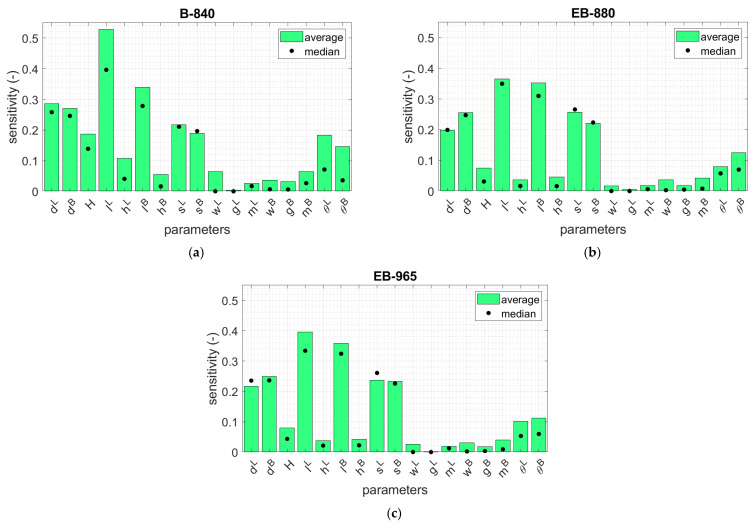
Average and median sensitivity for 20 cases of open-top boxes for the storage of fruits considered for different cardboards: (**a**) B-840, (**b**) EB-880 and (**c**) EB-965.

To compare which parameters were the most significant in terms of their compressive strength capacity, the average sensitivity of each parameter was determined from 20 geometries. In Figure 8, the averaged values of the sensitivities for three corrugated cardboards are presented and sorted in ascending order. Two horizontal dashed lines indicate levels of 0.05 and 0.10. In addition, in Figure 9, the average sensitivity values of the three corrugated boards used are shown by bar plots. The median of each parameter is also marked with black dots.

## 4. Discussion

All crucial results have been presented in Figure 6, Figure 7, Figure 8 and Figure 9 and Table 3. Considering Figure 6, in which the maximal displacement values have been presented for the selected cases, it may be observed that the buckling modes are not completely repeatable when compared case to case. Namely, in Figure 6a, the maximal displacements are obtained for shorter sidewalls, while Figure 6b,c shows the opposite situation, i.e., the maximal displacements are acquired for longer sidewalls. It can be concluded that the stiffness of the sidewalls plays a role, which, in a modifiable range of parameters, can switch the maximum buckling displacement between shorter or longer sidewalls. Moreover, regardless of the stiffness ratio of the shorter/longer sidewalls, the buckling mode in the hypotenuse demonstrates two half-waves, which is observed in all cases (see Figure 6). Shorter and longer sidewalls show one half-wave between the boundary conditions. In addition, it is worth noting that the buckling modes were obtained by applying rotations (see Section 2.2); therefore, here, the maximal displacement values are not equal to one.

Considering Figure 7, in which the effective Huber–Mises-Hencky stresses have been presented for the selected cases, it may be observed that the maximal stresses were obtained at the base of the hypotenuse, in which there is a complex stress state. In addition, higher stresses are visible in the upper part of the shorter sidewalls in the gluing zones with stiffening columns, which is particularly visible in Figure 7c. Moreover, all fields of effective stresses show that the highest values are obtained in the stiffening corners, which was expected. However, it seems that the position and shape of the edge holes relative to the arms of the trapezoidal folds affect the stress trajectories of the sidewalls; we acquire different stress zones in the sidewalls depending on the position of the edge holes. This can be seen by comparing the green and blue areas in Figure 7.

In this study, the sensitivities were computed for all the materials considered, namely, B-840, EB-880, and EB-965. However, the detailed tabular values were presented only for the case of B-840 case (see Table 3). The reason for this is the high similarity of the obtained results, as evidenced by the synthetic data shown for all materials in the bar graphs in Figure 9. The results show that $d^{L}$ and $d^{B}$ have a large influence on BCT, for which, on average, the sensitivity was 0.25 for the cardboard types (see Figure 8 and compare with Table 3). The longer these dimensions, the stiffer the corners, but the buckling lengths increase. Furthermore, the box height H has lower non-negligible impact because the average sensitivity for all cardboard types was 0.12. The lengths of the sidewall folds, $l^{L}$ and $l^{B}$, have the largest influence on the change in the BCT (0.39, on average, for the cardboard types): their decrease increases the strength of the material that is bearing the compression load in the corners. Two other parameters, the sides of the stiffening triangles, $s^{L}$ and $s^{B}$, play a minor role, but are still very important compared to other parameters. In the case of $s^{L}$, the average sensitivity for all cardboards was 0.24, while in the case of $s^{B}$, the average sensitivity for all cardboards was 0.21. The larger the triangular sides, the stronger the stiffening corner; its buckling length of the hypotenuse will also increase. The height of the folds of the sidewalls, $h^{L}$ and $h^{B}$, have a minor impact on the BCT, which can be regarded as negligible (average sensitivity for material parameters was 0.055). As suspected by engineering intuition, but now confirmed by the sensitivity study, some parameters related with the dimensions and positions of edge holes can be neglected (see Table 3 and Figure 8); these parameters are $w^{L}$, $g^{L}$, $m^{L}$, $w^{B}$, $g^{B},$ and $m^{B}$. These parameters represent the dimensions of the edge holes (width and height), as well as their positions from the perpendicular sidewalls in both types of sidewalls (shorter and longer). The inclination of the arms of the trapezoidal folds, i.e., $\theta_{L}$ and $\theta_{B}$, are more important, with an average sensitivity of 0.13. It is worth noting that the conclusion regarding the lack of influence of the size of the ventilation holes on the load capacity of the package is true only when the strength test of the tray is performed using a BCT press. Under natural operating conditions, when a tray is placed on top of another tray, the load on the bottom of the tray becomes significant. This means that the standard for assessing the load capacity of the tray should be different, e.g., considering the load on the bottom of the tray and other boundary conditions (i.e., supports should be applied only at the corners).

The averaged values for all the parameters considered are presented in Figure 8. Seven of the seventeen parameters were observed to have a sensitivity equal to or less than 0.05 ($w^{L}$, $g^{L}$, $m^{L}$, $w^{B}$, $g^{B}$, $m^{B},$ and $h^{B}$). These parameters can be assumed as constants by default when making decisions regarding the design of an optimal open-top box for the storage of fruits and vegetables in accordance with the BCT test. If a sensitivity value equal to 0.10 is taken as a threshold, the $h^{L}$ parameters can also be omitted.

## 5. Conclusions

In this paper, a parametric model of an open-top, corrugated board carton was built. Several geometrical parameters of box design were specified, and in order to check the impact of each of them on the compression strength of the packaging, a systematic sensitivity analysis was carried out. Several dozen sets of parameters were adopted for the proper exploration of parameter space. In the first step of the simulations, a buckling analysis was performed; then, the actual compression of the box was simulated. The load capacities and the sensitivities to the perturbation of each parameter were obtained from the computations. Based on the analyses carried out, the parameters were ranked in terms of importance, and several of them were deemed to be insignificant in terms of their impact on compressive strength based on the BCT test. In contrast, some of them play a very important role in the construction of the packaging and have a large impact on its load capacity; these parameters should be selected very carefully in the process of the optimal design of trays for containing vegetables or fruit.

In particular, this paper shows that not all geometric features are important from the perspective of compressive strength. The dimensions and location of the ventilation holes on both sidewalls, as well as the height of the trapezoidal folds on the shorter sidewalls, played minor roles in the load-bearing capacity of the packaging in a BCT-based testing protocol. For this reason, the indicated parameters may be assumed as constants and be neglected in the parametric model. Such selection is particularly useful for creating a reduced parametric model for use in an artificial neural network. Limiting the number of parameters allows one to save computational time and more effectively explore the space of crucial parameters in ANN optimization problems.

## Figures and Tables

**Table 1:** Geometric parameters of selected cases of open-top boxes; all dimensions, apart from the last two columns, are shown in mm.

Box Case	$d^{L}$	$d^{B}$	H	$l^{L}$	$h^{L}$	$l^{B}$	$h^{B}$	$s^{L}$	$s^{B}$	$w^{L}$	$g^{L}$	$m^{L}$	$w^{B}$	$g^{B}$	$m^{B}$	$\theta^{L}$ (°)	$\theta^{B}$ (°)
1	366	282	94	274	30	141	37	36	31.5	25	12.5	134.5	25	12.5	91.5	33.5	65
2	330	282	94	238	30	141	37	36	31.5	25	12.5	134.5	30	15	91.5	33.5	65
3	366	254	94	274	30	113	37	36	31.5	30	15	134.5	25	12.5	91.5	33.5	65
4	366	282	85	274	30	141	37	36	31.5	25	12.5	90	25	12.5	70	33.5	65
5	366	282	94	214	30	141	37	36	31.5	20	10	114.5	20	10	111.5	90	90
6	366	282	94	220	30	141	37	50	40	32	16	134.5	36	18	91.5	33.5	65
7	386	282	94	220	30	141	37	30	40	32	16	134.5	25	12.5	91.5	45	45
8	386	302	94	220	40	141	40	30	40	32	16	134.5	25	12.5	91.5	75	60
9	391	262	104	220	40	141	40	25	25	28	14	125	25	12.5	91.5	75	60
11	396	262	90	220	40	141	40	45	32	35	14	125	30	12.5	91.5	80	85
12	401	265	98	240	20	161	20	38	40	34	17	125	28	14	91.5	55	45
13	386	268	98	186	20	101	20	38	32	20	10	125	20	10	91.5	55	45
14	388	271	98	206	15	121	25	42	45	20	10	155	20	10	111	55	45
15	392	252	94	206	15	121	25	30	27	20	10	92	20	10	85	55	45
16	396	252	81	300	15	170	25	30	27	20	10	135	20	10	111	20	35
17	398	252	81	300	35	170	35	30	27	20	10	135	20	10	111	37	35
18	396	252	83	261	25	150	35	36	36	35	10	135	35	10	85	65	55
19	310	290	85	174	25	150	35	30	28	16	8	95	16	8	85	65	55
20	320	271	81	174	25	160	35	40	28	22	11	115	22	11	95	40	45

**Table 2:** Material data used in the constitutive models of corrugated boards considered herein.

Grade	$E_{1}$	$E_{2}$	$\nu_{12}$	$G_{12}$	$G_{13}$	$G_{23}$	$\sigma_{0}$	$R_{11}$
	(MPa)	(MPa)	(–)	(MPa)	(MPa)	(MPa)	(MPa)	(–)
B-840	2032	1111	0.40	1184	7	11	3.05	0.95
EB-880	1636	907	0.40	963	8	11	3.50	0.65
EB-965	1616	750	0.44	898	7	11	3.01	0.74

**Table 3:** Sensitivities computed for B-840 corrugated cardboard with the min/max values marked in blue and red for all parameters considered in the study.

Case	$d^{L}$	$d^{B}$	H	$l^{L}$	$h^{L}$	$l^{B}$	$h^{B}$	$s^{L}$	$s^{B}$	$w^{L}$	$g^{L}$	$m^{L}$	$w^{B}$	$g^{B}$	$m^{B}$	$\theta^{L}$	$\theta^{B}$
1	−0.10	0.31	−0.01	−1.23	−0.30	−0.30	0.01	0.14	0.21	0	0	0	0	0	−0.08	−0.25	0.02
2	0.18	0.55	−0.02	−1.17	−0.38	−0.49	−0.17	0.22	0.03	0	−0.04	−0.17	−0.06	−0.06	−0.19	−0.29	−0.04
3	−0.04	0.33	0.12	−1.31	−0.31	−0.25	0.13	0.23	0.22	0	0	0	0.09	0.09	0.03	−0.17	0.15
4	0.22	−0.67	0.20	−0.57	0	−0.39	0.02	0.23	0.21	0	0	0.01	−0.01	−0.01	−0.02	0.08	−1.25
5	0.29	0.29	0.06	−0.45	−0.05	−0.29	−0.01	0.21	0.20	0	0	0	0	0	0	0.12	0.14
6	0.19	0.06	0.15	−0.28	0.01	−0.22	0	0.25	0.18	0	0	0.01	−0.01	−0.02	0.04	0.01	0.03
7	0.25	0.16	0.13	−0.33	0.02	−0.25	0	0.16	0.27	0	0	0.02	0.01	0.01	0.02	0.07	0.02
8	0.27	0.11	0.09	−0.37	−0.01	−0.23	0.01	0.11	0.22	−0.02	−0.02	−0.02	0	0	0.01	0	−0.01
9	0.55	0.31	−0.38	−0.46	−0.02	−0.27	0.06	0.17	0.19	0	0	−0.02	0	0.05	0.05	0	0.07
10	0.36	0.24	0.13	−0.33	0.02	−0.29	0	0.26	0.18	0	0	0	0	0	0.07	0.10	0.11
11	0.29	0.24	0.14	−0.43	0.01	−0.95	0.01	−0.21	0.23	−0.70	0	0	−0.01	−0.01	0.01	−0.06	0.01
12	0.29	0.31	0.19	−0.22	0.05	−0.34	0.02	0.23	0.19	0.01	0	0	0	0	0.01	0.02	0.04
13	0.27	−0.08	0.13	−0.21	0.12	−0.17	−0.06	0.21	0.19	0	0	0	−0.35	0	−0.31	0	0.08
14	0.33	0.25	0.14	−0.33	0.04	−0.26	0.01	0.18	0.17	0	0	0.02	0	0	0	0.03	0.02
15	0.24	0.20	0.63	−0.76	0.12	−0.43	−0.10	0.23	0.26	0	0	0	−0.01	−0.01	0.02	0.12	0.02
16	0.19	0.21	−0.33	−0.76	0	−0.76	0	0.20	−0.03	−0.09	0	0.02	−0.02	−0.02	0.01	−0.06	0.02
17	0.25	0.48	0.35	−0.47	0.08	−0.15	0.27	0.20	0.24	0	0	−0.03	0	0.26	0.27	0.08	0.29
18	0.60	0.29	0.17	−0.24	0.44	−0.24	0.07	0.17	0.20	0.38	0	−0.03	0.07	0	0.07	0.36	0
19	0.23	−0.15	0.35	−0.35	0.18	−0.23	0.11	0.44	0.28	−0.07	0	−0.07	0.09	0.09	0.09	1.82	0.59
20	0.58	0.16	0.02	−0.28	0	−0.29	−0.04	0.28	0.11	0.01	0	−0.07	0	0	0.01	0.04	0.04

## Data Availability

The data presented in this study are available on request from the corresponding author.
